# Mitochondria in aging cell differentiation

**DOI:** 10.18632/aging.100993

**Published:** 2016-06-29

**Authors:** Zdena Palková, Libuše Váchová

**Affiliations:** Department of Genetics and Microbiology, Faculty of Science, Charles University in Prague, Prague, Czec Republic

**Keywords:** mitochondria, cell differentiation, retrograde signaling, aging, yeast colonies, cancer

Mitochondria, key organelles of eukaryotic cells that are responsible for essential metabolic processes, generation of energy, cellular redox state and many other processes, are deeply involved in cellular homeostasis and influence overall cell/organism physiology. Defects in mitochondria can lead to a number of different disorders in mammals [1]. Changes in the functional state of mitochondria can induce cellular responses via activation of “mitochondrial retrograde signaling” pathway(s). Such responses often result in changes in gene expression and overall cell physiology leading to the prevention of cell death, as described in mammals and yeast [2]. In mammals, physiological changes induced by retrograde signaling are often linked to cancer-related disorders, including the activation of different oncogenic factors and enzymes involved in aerobic glycolysis [1]. The major pathway involved in retrograde signaling in yeast (the RTG pathway) is mediated by three activators Rtg1p, Rtg2p and Rtg3p. Rtg2p transfers the signal from mitochondria to the Rtg1p/Rtg3p heterodimeric transcriptional activator that translocates from cytosol to nucleus and activates expression of numerous genes involved in yeast metabolic reprogramming. The RTG pathway is negatively regulated by Mks1p and Bmh1p/2p. TORC1 negative regulation has also been observed [3]. The activation of anaplerotic reactions and peroxisomal functions, including the glyoxylate cycle, has long been considered to be a major RTG pathway response in yeast and the *CIT2* gene, encoding the peroxisomal isoform of citrate synthase, as a typical target of the RTG pathway.

**Figure 1:**
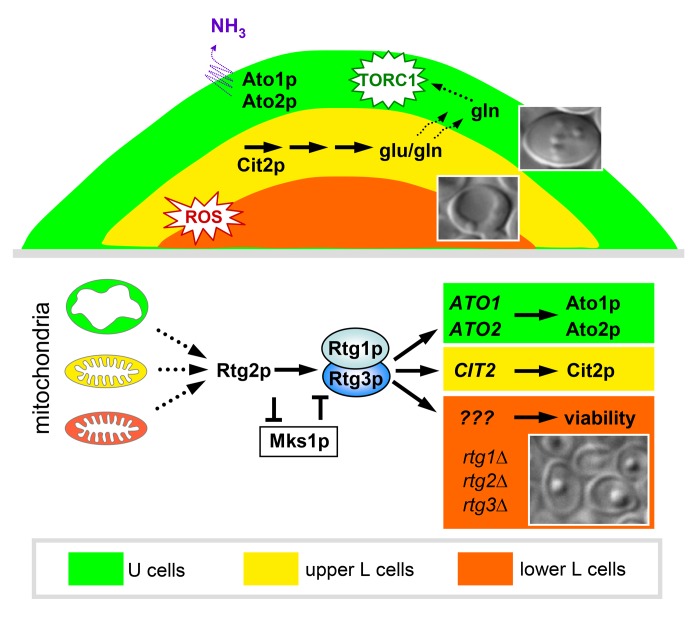
Model scheme displaying three branches of RTG signaling involved in yeast colony differentiation and formation of the three specifically localized cell subpopulations as schematically shown in vertical colony cross-section.

Recently, we have provided evidence that the RTG response in yeast is more complex than previously assumed, involves a number of yet to be identified regulatory elements and affects different cellular processes and the subsequent fate of differentiated yeast cells [4]. In differentiated yeast colonies we have identified 3 different branches of RTG signaling, regulated by differently altered mitochondria and leading to expression of different gene targets and thus to divergent metabolic reprogramming. These findings build upon our previous identification on two major cell types that develop within ageing, differentiated colonies, regulated by ammonia signaling - vital U cells in upper colonial regions that gain unique metabolic properties important for the longevity of these cells and starving, respiration-competent L cells in lower regions that provide nutrients to U cells [5, 6]. Dampened, ROS-free mitochondria activate the “Ato-branch” of RTG signaling in modestly respiring U cells, leading to the activation of expression of *ATO1* and *ATO2* genes, involved in ammonia production and metabolic reprogramming of these cells [4]. Contrary to previous reports, describing negative regulation of RTG signaling by TORC1, the “Ato branch” is active in parallel with active TORC1 in U cells [5, 7]. Two other RTG signaling branches activate different processes in two subpopulations of L cells, i.e., in cells with inactive TORC1. The “Cit2p-branch” is active in upper L cells and activates *CIT2* expression and related metabolic reprogramming that may lead to production of glutamate/glutamine potentially released from these L cells and consumed by neighboring U cells. Increased intracellular glutamine concentration and/or glutaminolysis could then be involved in the activation of TORC1 signaling in U cells. In lower L cells the functionality of RTG signaling is essential for a viability of these cells, therefore we call this branch the “viability branch”. Neither *ATO* nor *CIT2* genes are expressed in lower L cells. Importantly, all three of the Rtg activators, as well as the Mks1p repressor, are essential for each of the three branches of RTG signaling, despite the fact that these branches lead to expression of different gene targets and affect different cellular processes (Figure 1). These findings indicate that as-yet unidentified co-activators/co-repressors of Rtg regulators likely exist that are specific to particular branches of RTG signaling. In addition, the fact that U and L cells gain differently altered mitochondria - swollen dampened mitochondria in U cells versus respiratory competent mitochondria with increased reactive oxygen species (ROS) in L cells - suggests the intriguing possibility that differential mitochondrial status is involved in the specification of a particular branch of RTG signaling. In other words, these data imply that mitochondria can enter different states, which can divergently affect subsequent cellular development.

The observed heterogeneity of RTG signaling within yeast colonies, that contributes to diversification of specifically localized cell subpopulations, resembles the diversity of mitochondrial retrograde signaling in mammals, which includes a number of regulatory events under different conditions and in different cells. This signaling is regulated through a variety of largely unknown factors. Future identification of new upstream and downstream elements involved in the regulation of RTG signaling in specialized cell types, developing within relatively simple yeast colonies that metabolically resemble tumor-affected organisms, may thus facilitate the identification of similar elements of retrograde signaling involved in cellular differentiation in mammals.
